# Functional Characterization of Antithrombin Mutations by Monitoring of Thrombin Inhibition Kinetics

**DOI:** 10.3390/ijms22042119

**Published:** 2021-02-20

**Authors:** Sara Reda, Jens Müller, Anna Pavlova, Behnaz Pezeshkpoor, Johannes Oldenburg, Bernd Pötzsch, Heiko Rühl

**Affiliations:** Institute of Experimental Hematology and Transfusion Medicine, University of Bonn, Venusberg-Campus 1, 53127 Bonn, Germany; sara.reda@ukbonn.de (S.R.); jens.mueller@ukbonn.de (J.M.); Anna.Pavlova@ukbonn.de (A.P.); Behnaz.Pezeshkpoor@ukbonn.de (B.P.); Johannes.Oldenburg@ukbonn.de (J.O.); Bernd.Poetzsch@ukbonn.de (B.P.)

**Keywords:** thrombin, antithrombin deficiency, SERPINC1 gene, coagulation inhibition

## Abstract

Inactivation of thrombin by the endogenous inhibitor antithrombin (AT) is a central mechanism in the regulation of hemostasis. This makes hereditary AT deficiency, which is caused by SERPINC1 gene mutations, a major thrombophilic risk factor. Aim of this study was to assess to what extent AT mutations impair thrombin inhibition kinetics. The study population included 36 thrombophilic patients with 19 different mutations and mean AT levels of 65% in a thrombin-based functional assay, and 26 healthy controls. To assess thrombin inhibition kinetics, thrombin (3.94 mU/mL final concentration) was added to citrated plasma. Subsequently, endogenous thrombin inhibition was stopped by addition of the reversible thrombin inhibitor argatroban and the amount of argatroban-complexed thrombin quantified using an oligonucleotide-based enzyme capture assay. The plasma half-life of human thrombin was significantly longer in patients with AT mutations than in the controls (119.9 versus 55.9 s). Moreover, it was disproportionately prolonged when compared with preparations of wild type AT in plasma, in whom a comparable thrombin half-life of 120.8 s was reached at a distinctly lower AT level of 20%. These findings may help to better understand the increased thrombotic risk of SERPINC1 mutations with near normal AT plasma levels in functional assays.

## 1. Introduction

The serine protease inhibitor (SERPIN) antithrombin (AT) is an important physiological anticoagulant protein. It inhibits clot formation by forming enzymatically inactive complexes with the active coagulation factors thrombin, activated factor X (FXa) and, less importantly, activated factors IX and XI [1,2]. The mechanism by which AT exerts its inhibitory function requires the interplay of two different regions of the protein: a reactive site that binds to the enzymatically active site of the target serine protease and a heparin-binding site. Through this heparin-binding site a conformational change is induced in the presence of heparin leading to a more than 1000-fold acceleration of AT activity [3].

Reflecting the key role of AT in the regulation of clot formation, hereditary AT deficiency is considered a “classical” thrombophilic risk factor and is associated with the highest risk of venous thromboembolism (VTE) among the hereditary thrombophilias [4]. The estimated prevalence of hereditary AT deficiency varies widely, with estimates between 1:500 and 1:5000 [5]. More than 250 mutations in the AT gene *SERPINC1* have been reported so far [6]. The risk of VTE is increased approximately 5- to 50-fold in individuals with hereditary AT deficiency. Clinical presentation and severity vary considerably depending on the underlying mutation, and do not always correlate with the concentration of the protein in the circulation as measured using functional assays [3,4].

The thrombin and FXa inhibiting properties of AT are utilized in commercially available assays for the measurement of AT levels in plasma. In these assays, bovine thrombin or FXa of either human or bovine origin are added in excess to diluted citrated plasma. After incubation, the residual thrombin or FXa activity is measured through cleavage rates of a peptide-specific chromogenic substrate. The plasma concentration of functionally active AT is inversely proportional to the measured absorbance rate [7]. Depending on the use of thrombin or FXa, bovine or human origin of utilized thrombin, or the presence of heparin in the reaction mixture, functional AT assays may yield different results, which in combination with AT antigen levels are characteristic for different AT mutations [3,5,8,9,10]. However, if and to what extent AT mutations affect thrombin or FXa inhibition kinetics has not been characterized so far.

In a previous study we have presented a novel approach to measure thrombin inactivation kinetics at sub-threshold thrombin concentrations [11]; plasma levels that are too low to induce fibrin clot formation but are high enough to induce a prothrombotic phenotype by activation of factors V, VIII, and XI [12]. This approach utilizes an oligonucleotide-based enzyme capture assay (OECA) that quantifies thrombin in plasma with a limit of quantification of 1.08 pmol/L [13]. We were able to show a clear dependence of thrombin inhibition kinetics on plasma levels of wild type AT [11]. In the present study we extended this approach to carriers of AT mutations in whom the inhibition kinetics of human and bovine thrombin were assessed. Compared with healthy controls the plasma half-life was significantly prolonged in AT mutation carriers. Most importantly, this prolongation of thrombin half-life distinctly exceeded that of wild type plasma preparations of equivalent residual activity in a high number of AT mutations. In conclusion, the assessment of thrombin inhibition kinetics can be used to better characterize AT mutations and may provide a better understanding of the pathophysiological and clinical consequences of specific mutations.

## 2. Results

### 2.1. Study Population

The study population included 36 subjects (24 females) with a mean age of 35 (range: 4–82) years who had mutations of the *SERPINC1* gene. Twenty-three (64%) had a history of venous or arterial thrombosis while the others were asymptomatic. The most prevalent gene variant in the study population was c.391C>T (p.Leu131Phe, “AT Budapest III”) [14] with 11 carriers, thereof five homozygous children of two heterozygous parents. The study population included three subjects each who were heterozygous carriers of the gene variants c.89T>A (p.Val30Glu, “AT Dublin”) [15], c.462_464del (p.Phe155fs) [16], and c.1153G>A (p.Gly385Ser). Furthermore, two heterozygous carriers of c.1058C>T (p.Pro353Leu) [17] and one heterozygous carrier each of 14 other different mutations were included. In all 36 subjects with *SERPINC1* mutations functional AT levels (using thrombin-based and FXa-based assays) and AT antigen levels were determined in addition to the assessment of the inhibition kinetics of human thrombin. The applied thrombin-based assay utilizes bovine thrombin, in order to render the interference of heparin cofactor II insignificant [18,19]. Therefore, to allow for a better comparison of measurements of thrombin inhibition and functional AT levels, we also assessed the inhibition kinetics of bovine thrombin in a subset of 19 *SERPINC1* mutation carriers, of whom we had sufficient sample material at our disposal. Table 1 summarizes the characteristics of the study subjects with *SERPINC1* mutations. A complete list of patients with characteristics and measurement results on an individual basis is provided in the Supplementary Materials (Table S1).

Inhibition kinetics of human thrombin were additionally assessed in a control group of healthy blood donors which included 26 subjects (thereof 10 females) with a mean age of 35 (range: 22–59) years.

### 2.2. Thrombin Inhibition Capacity Is Reduced in Most Patients with Antithrombin Mutations

**Figure 1:**
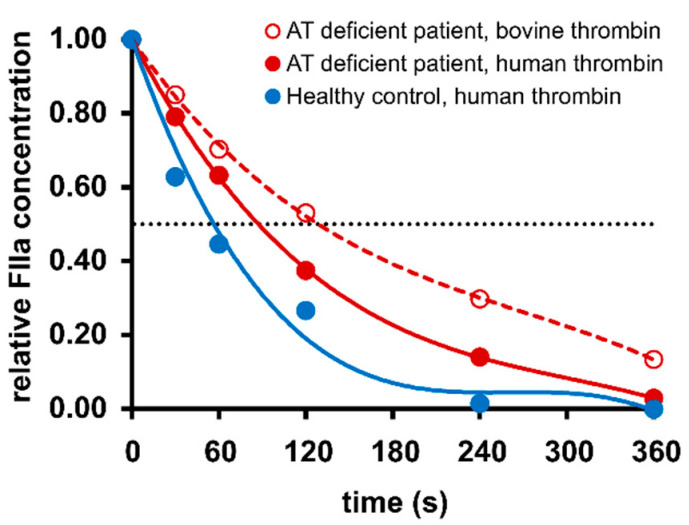
Inactivation kinetics of thrombin in representative human plasma samples. Purified thrombin (FIIa) was added to citrate-anticoagulated plasma obtained from a healthy individual (human thrombin, blue circles) or a patient with hereditary antithrombin (AT) deficiency (human thrombin, red filled circles; bovine thrombin, red unfilled circles) to achieve a final concentration of 2 ng/mL. At the indicated time points, thrombin inactivation was stopped by the addition of argatroban at a final concentration of 200 µmol/L and the residual thrombin activation measured using the thrombin-oligonucleotide-based enzyme capture assay.

Figure 1 shows typical inhibition curves of exogenously added human thrombin in citrated plasma obtained from a healthy individual and a patient with the *SERPINC1* mutation c.462_464del (p.Phe155fs), and the inhibition curve of bovine thrombin in plasma of the same patient.

**Figure 2:**
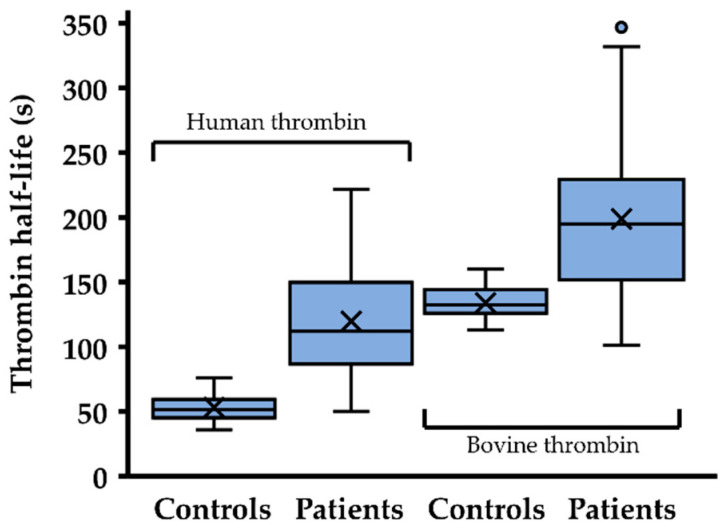
Inactivation rates of thrombin in healthy individuals and antithrombin (AT)-deficient patients. The half-life of exogenously added thrombin (2 ng/mL final concentration) was determined in healthy controls (*n* = 26) and patients with AT mutations (*n* = 36, human thrombin; *n* = 19, bovine thrombin). The boxes show quartiles and the median of the data, the whiskers extend up to 1.5 times of the interquartile range from the box, and circles show outlying data values. X indicates the mean.

The mean plasma half-life of human thrombin was significantly prolonged in the cohort with AT mutations in comparison to the control group, with 119.9 ± 41.0 vs. 55.9 ± 9.6 s (*t* test *p* = 2.8 × 10^–11^) but it was shorter than the plasma half-life of bovine thrombin in patients with AT mutations (198.9 ± 63.6 s, *t* test *p* = 1.3 × 10^–4^) (Figure 2). 

Table 2 shows the plasma half-life of thrombin in the different AT mutations prevalent in our study population. In three patients with three different *SERPINC1* mutations (c.74G>A, c.236G>A, c.1246G>T) a plasma half-life of human thrombin within the lower 95th percentile of the control group (73.2 s) was observed. In the other AT mutations, the plasma half-life of human thrombin exceeded this value. In every AT mutation a longer plasma half-life of bovine thrombin than of human thrombin was observed. The plasma half-lives of human and bovine thrombin showed a strong correlation to each other (Pearson’s correlation coefficient *r* = 0.599, *p* = 0.007).

### 2.3. Influence of Antithrombin Level on Thrombin Inhibition Kinetics

In order to study how changes in the plasma level of wild type AT influence thrombin inhibition kinetics, normal pooled plasma (NPP) was mixed with AT-deficient plasma and thrombin inhibition kinetics measured. A mean plasma half-life of human thrombin of 58.6 ± 10.7 s measured at 100% thrombin-based AT activity (102% FXa-based AT activity, 100% AT antigen level) in undiluted NPP prolonged to a maximum of 156.6 ± 25.0 s in AT-deficient plasma. The mean plasma half-life of bovine thrombin was 120.6 ± 24.0 s in undiluted NPP and 514.3 ± 106.6 s in AT-deficient plasma.

**Figure 3:**
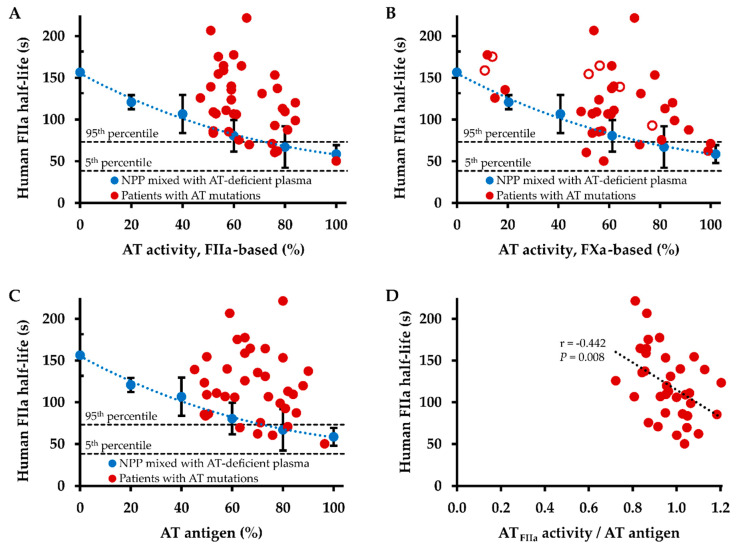
Dependence of inhibition kinetics of human thrombin (FIIa) on antithrombin (AT) plasma levels. (**A**) The half-life of exogenously added human thrombin (2 ng/mL final concentration) was determined in citrated plasma obtained from patients with AT mutations (red circles, *n* = 36)) and in normal pooled plasma (NPP) mixed with AT-deficient plasma to achieve the indicated AT levels (blue circles). Data points in blue represent means of three independent determinations on three different days, error bars represent the standard deviation. The intersected lines parallel to the x-axis indicate the 95th and 5th percentiles of half-life times measured in healthy controls (*n* = 26). Functional AT levels were determined by a thrombin-based assay. (**B**) Functional AT levels were determined by an activated factor X (FXa)-based assay. Data points filled white indicate plasma from patients under therapy with FXa inhibitors. (**C**) AT antigen levels were determined by an immunoassay. (**D**) Correlation of the ratio of AT activity determined by a FIIa-based assay and AT antigen concentration with the plasma half-life of exogenously added human thrombin. r indicates the Pearson correlation coefficient.

Figure 3 shows the dependence of the plasma half-lives of human thrombin in AT mutations and wild type AT on thrombin-based AT activity (Figure 3A), FXa-based AT activity (Figure 3B), and AT antigen level (Figure 3C). Plasma half-lives in patients with AT mutations were comparable with those in NPP preparations with equivalent AT plasma levels, or remarkably, were distinctly longer. This pattern was more clearly found for thrombin-based AT activity and AT antigen levels, whereas two patients with AT mutations (c.236G>A and c.391C>T) showed markedly shorter half-lives than expected for their FXa-based AT activity levels of 58% and 51%, respectively. With 120.8 s at 20% thrombin-based AT activity, the mean plasma half-life of human thrombin in the NPP preparations corresponded to that in patients with AT mutations (119.9 s), in whom a mean thrombin-based AT activity of 65% was observed.

While there was a tendency to longer plasma half-lives of human thrombin with lower AT levels, the correlations of plasma half-life with thrombin-based AT level, FXa-based AT level, and AT antigen level did not reach statistical significance. However, there was a strong negative correlation between the ratio of thrombin-based AT activity level and AT antigen level with the plasma half-life of human thrombin (*r* = −0.442, *p* = 0.008, Figure 3D). The ratio of FXa-based AT activity level and AT antigen level correlated moderately negative with the plasma half-life of human thrombin (Spearman’s rank correlation coefficient *r*_s_ = −0.334, *p* = 0.046).

**Figure 4:**
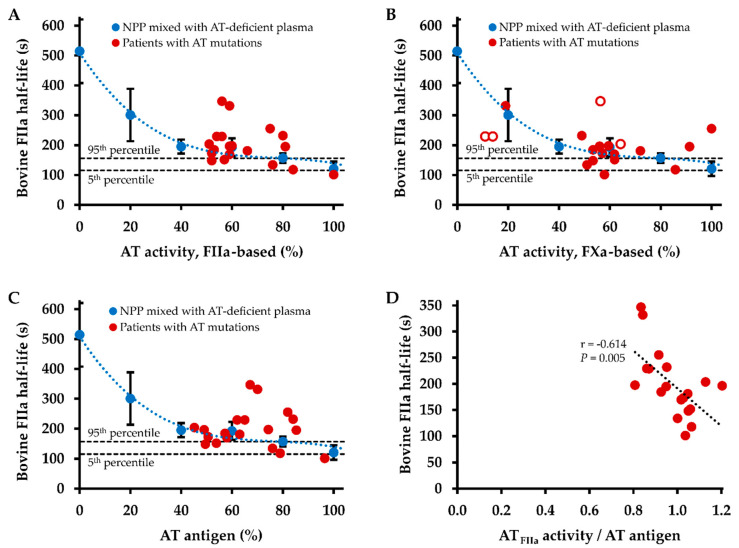
Dependence of inhibition kinetics of bovine thrombin (FIIa) on antithrombin (AT) plasma levels. (**A**) The half-life of exogenously added bovine thrombin (2 ng/mL final concentration) was determined in citrated plasma obtained from patients with AT mutations (red circles, *n* = 19) and in normal pooled plasma (NPP) mixed with AT-deficient plasma to achieve the indicated AT levels (blue circles). Data points in blue represent means of three independent determinations on three different days, error bars represent the standard deviation. The intersected lines parallel to the x-axis indicate the 95th and 5th percentiles of half-life times measured in healthy controls (*n* = 26). Functional AT levels were determined by a thrombin-based assay. (**B**) Functional AT levels were determined by an activated factor X (FXa)-based assay. Data points filled white indicate plasma from patients under therapy with FXa inhibitors. (**C**) AT antigen levels were determined by an immunoassay. (**D**) Correlation of the ratio of AT activity determined by a FIIa-based assay and AT antigen concentration with the plasma half-life of exogenously added bovine thrombin. r indicates the Pearson correlation coefficient.

Figure 4 correlates the plasma half-life of bovine thrombin in AT mutations and wild type AT with thrombin-based AT activity (Figure 4A), FXa-based AT activity (Figure 4B), and AT antigen level (Figure 4C). Similar with the pattern observed for the inhibition of human thrombin, plasma half-lives of bovine thrombin were distinctly longer in several patients with AT mutations compared with those in NPP preparations with equivalent thrombin-based AT activity and AT antigen levels. However, this pattern was less pronounced for bovine thrombin than for human thrombin: The mean ratio of the plasma half-life of human thrombin in patients with AT mutations and the estimated plasma half-life of wild type AT was 1.51 ± 0.48. For the plasma half-lives of bovine thrombin this ratio was smaller, with 1.16 ± 0.36 (*t* test, *p* = 0.008).

There was no statistically significant correlation of the plasma half-life of bovine thrombin with thrombin-based AT level, FXa-based AT level, AT antigen level, and the ratio of FXa-based AT level and AT antigen level. There was a strong negative correlation between the ratio of thrombin-based AT activity level and AT antigen level with the plasma half-life of bovine thrombin (*r* = −0.614, *p* = 0.005, Figure 4D).

## 3. Discussion

The thrombin inhibition capacity was impaired in plasma of patients with AT mutations compared with plasma from healthy controls, as demonstrated by a significantly prolonged thrombin half-life. If the lower 95th percentile of thrombin half-lives in the control group is taken as reference, three AT mutation carriers with different mutations which were not present in other study subjects, showed no prolonged half-life of human thrombin. The shortest thrombin half-lives were observed in a patient with a c.236G>A mutation (p.Arg79His, “AT Rouen”), which is located at the heparin binding site [20] and another patient with a c.1246G>T mutation (p.Ala416Ser, “AT Cambridge II”) at the reactive site [21]. These findings were consistent with observed normal or borderline reduced thrombin-based AT activity levels of 100% and 77%, respectively, in these patients, which are in accordance with the literature [20,21,22]. The third patient with an AT mutation without prolonged thrombin half-life, c.74G>A (p.Gly25Asp), showed clearly reduced thrombin-based AT activity (66%) and antigen (63%) levels. Vice versa, there were two patients with two different AT mutations, c.1347del G (p.Leu449fsX9) and c.1355T>C (p.Ile452Thr), in whom normal thrombin-based activity levels in combination with prolonged human thrombin half-life times were measured. In a previous study from our institution that first described the c.1347del G mutation, an AT activity of 57% was reported in a carrier of this mutation [16].

It must be noted that the afore-mentioned discrepancies between thrombin-based AT activity levels and plasma half-life times of human thrombin were observed in AT mutations that were present only in individual study subjects and therefore prone to outlying measurements of one or both parameters. In addition to these singular cases, two of six heterozygous carriers of the heparin-binding site mutation AT Budapest III [14,23] with borderline reduced thrombin-based AT activity (75% and 76%) showed a normal plasma half-life of human thrombin (71.0 s and 60.6 s). However, in the overall cohort of heterozygous carriers of AT Budapest III the plasma half-life of human thrombin was abnormally prolonged and lay above the lower 95th percentile of the control group. The plasma half-life of human thrombin in all other study subjects was prolonged according to the same definition, despite the heterogeneity of the AT mutations present in the study population.

In addition to AT, the structurally similar SERPIN heparin cofactor II plays a significant role in the inhibition of thrombin, especially in the presence of heparin and under conditions, when AT is decreased [18,24]. Plasma levels of heparin cofactor II show considerable intra-individual variation and appear to be modulated by various factors including age, nutrition, and estrogen-containing drugs [25,26]. In order to minimize a potential interfering effect of heparin cofactor II with thrombin inhibition kinetics, we conducted all thrombin half-life measurements in the absence of heparin and we additionally measured the half-life of bovine thrombin in plasma, if enough sample material was available. It has been shown that heparin cofactor II interferes with the inhibition of bovine thrombin in human plasma only at very low AT levels [18,19]. With thrombin-based AT activity levels of more than 45% in all plasma samples from patients with AT mutations an interfering influence of heparin cofactor II on the inhibition kinetics of bovine thrombin can most likely be excluded. In the subgroup of patients with AT mutations, in whom the plasma half-life of bovine thrombin was assessed, it was significantly longer than that of human thrombin in the cohort as well as in every individual study subject. The strong correlation between human and bovine thrombin half-life times suggests, that the observed plasma half-life times of human thrombin are characteristic for the AT mutations, whose effects on both human and bovine thrombin half-life could be assessed. Taken together, our data indicate that an impaired thrombin inhibition capacity is a common finding in patients with AT mutations.

In a previous study we have already shown the dependence of the plasma half-life of human thrombin on the concentration of wild type AT [11]. Thus, the question arises if and what additional characterizing information on AT mutations can be obtained by the assessment of the thrombin inhibition kinetics. In contrast to the previously reported data on wild type AT, AT activity (either thrombin- or FXa-inhibition based) and antigen levels did not correlate significantly with the plasma half-life of human or bovine thrombin in carriers of AT mutations in the present study. Hence, thrombin half-life does not simply reflect (thrombin-based) functional AT levels, which is also indicated by disproportionately prolonged thrombin half-lives in relation to AT levels in several cases. For example, the three type I mutations c.462_464del (p.Phe155fs) [16], c.481C>T (p.Arg161Ter) [27], and c.379T>C (p.Cys127Arg, “AT Morioka”) [28] showed virtually identical thrombin-based AT activity levels of 51–52%, whereas the plasma half-lives of human thrombin were 92.3, 139.2, and 206.6 s, respectively. This disproportionate prolongation of the plasma half-life was also observed for bovine thrombin, e.g., 203.8 s in c.481C>T vs. 168.5 s in c.462_464del, ruling out the bovine origin of thrombin utilized in the thrombin-based AT activity assay as a potential cause of the observed reaction pattern. The disproportional prolongation of thrombin half-life in AT mutation carriers also becomes clear through the comparison with a dilution series of wild type AT. The ratio between thrombin half-life in AT mutation carriers and thrombin half-life in wild type AT plasma with equivalent thrombin-based AT activity levels was significantly smaller for bovine than for human thrombin, with 1.51 vs. 1.16. This can be explained by the insensitivity of bovine thrombin towards human heparin cofactor II, which results not only in longer plasma half-lives of bovine compared with human thrombin, but also reduces the variability in bovine thrombin half-life.

There are two possible approaches to explain the disproportionate prolongation of thrombin half-lives in AT mutations in relation to AT levels. First, the use of excess amounts of thrombin or FXa in functional AT assays may cover up relatively small effects of certain AT mutations on the interaction with thrombin or FXa, thus leading to an overestimation of residual functional AT activity levels. Second, in the case of heterozygous AT mutations the binding of functionally less active mutant protein to its reaction partners thrombin and FXa could compete with the binding of functionally normal wild type AT, thus additionally impairing the thrombin- or FXa-inhibiting capacity of plasma. This second proposed mechanism is strongly supported by our finding of a highly significant negative correlation between the ratio of AT activity/AT antigen level and thrombin half-life. However, it does not explain the disproportionately long thrombin half-life in homozygous carriers of AT Budapest III or in the patient with the large deletion which affected all exons of *SERPINC1*, whose plasma supposedly contain only mutant or only wild type AT, respectively. It is also conceivable that both proposed explanations apply simultaneously and/or at different extent, depending on specific AT mutations.

In our previous study we have shown that in vitro thrombin generation parameters correlate with the plasma half-life of thrombin [11]. One might therefore speculate if the thrombin half-life more precisely predicts the thrombotic risk than residual AT activity in AT mutations. Although an answer to this question is beyond the scope of this study, the disproportionate prolongation of the thrombin half-life in AT mutations with only slightly reduced AT levels underlines the high impact of these mutations on the overall thrombin-inhibiting capacity.

Potential sources of bias or imprecision in this study include the size and composition of the study population and the selection and conduction of laboratory tests. The prevalence of AT mutations in the study population was not representative and most mutations were only present in a single study subject. Furthermore, plasma half-live of bovine thrombin could not be measured in the whole cohort of patients with AT mutations. The commercially available tests used for the measurement of AT levels were also not representative and choosing other or additional tests might have resulted in different reaction patterns in specific AT mutations. Pre-analytical conditions, variability, and reproducibility of thrombin half-life measurement have been extensively assessed previously, using wild type AT [11]. To assess the precision of thrombin inhibition testing, coefficients of inter- and intra-assay variation (CV) have been determined previously: An intra-assay CV of 7.8% was determined by six simultaneous determinations of the same sample of NPP in one run. Analyzing NPP in a total of 17 independent runs revealed an inter-assay CV of 19.2% [11]. In the present study, the results obtained in healthy controls and in wild type AT preparations were in line with previously obtained data. However, it cannot be ruled out that variability and reproducibility of thrombin half-life measurements might be different in plasma from patients with AT mutations. A potential alternative normalization strategy, that would render the comparison to a plasma dilution series of wild type AT unnecessary, could be adjusting the AT antigen levels of every sample by addition of AT-deficient plasma before measuring thrombin half-life times.

In conclusion, the data obtained in this study show that thrombin inhibition kinetics are affected by AT mutations, as evidenced by the prolonged plasma half-life of thrombin in most of the assessed samples. In addition to the measurement of AT activity and antigen levels, assessment of thrombin inhibition kinetics in patients with hereditary AT deficiency can provide information to better characterize different AT mutations. The finding of disproportionately prolonged thrombin half-life times in relation to AT levels in several AT mutations warrants further research on a potential association between thrombin inhibition kinetics and thrombotic risk.

## 4. Materials and Methods

This study was conducted from April 2017 to October 2020 at the Institute of Experimental Hematology and Transfusion Medicine, Bonn, Germany. The study proposal was approved by the Institutional Review Board and Ethics Committee of the Medical Faculty of the University of Bonn on 17 May 2017 (protocol code 016/16). Written informed consent was obtained from all participants in compliance with the declaration of Helsinki. The procedures followed were in accordance with institutional guidelines.

### 4.1. Reagents and Materials

Human α-thrombin was obtained from CellSystems (St. Katharinen, Germany). Bovine α-thrombin and human AT-deficient plasma was purchased from Haematologic Technologies (Essex Junction, VT, USA). Citrated (10.5 mmol/L) platelet-poor NPP was prepared by mixing equal volumes of plasma obtained from healthy blood donors. A NPP preparation with AT levels most closely to 100% was selected for use in the experiments of this study. Argatroban (Argatra^®^) was obtained from Mitsubishi Pharma (Düsseldorf, Germany). General chemicals were purchased from Sigma-Aldrich (Munich, Germany). The 3′-biotinylated DNA-aptamer HD1–22 was synthesized and purified by Microsynth (Balgach, Switzerland).

### 4.2. Patients and Samples

From 1 April 2017 to 31 October 2020, blood samples were prospectively collected from patients who were referred to the outpatient clinic for coagulation disorders of our institution. Inclusion criteria were verified mutations in the *SERPINC1* gene. Patients receiving heparin or thrombin inhibitors were excluded. At the time of blood sampling 7 patients were under anticoagulant treatment with vitamin K antagonists and 6 received FXa inhibitors (Table S1). Blood samples from healthy controls and for the preparation of NPP were obtained from the blood donation service of our institution. All samples were obtained by puncture of a suitable vein using a 21G or 23G butterfly needle (Sarstedt, Nümbrecht, Germany). After discarding the first 2 mL, blood was drawn into citrate tubes (10.5 mM final concentration, Sarstedt, Nümbrecht, Germany) that were processed within 1 h of collection. Plasma was obtained by centrifugation at 3300× *g* for 10 min and aliquots stored at less than −70 °C until assaying. At the time of analysis, aliquots were thawed at 37 °C and analyses performed within 60 min after defrosting.

### 4.3. Laboratory Analysis of Blood Samples

Measurements of AT levels were conducted using the Atellica^®^ COAG 360 coagulation analyzer (Siemens Healthcare Diagnostics, Eschborn, Germany). AT activity levels were measured using one chromogenic substrate assays based on inhibition of thrombin (Berichrome AT III, Siemens Healthcare Diagnostics Products, Marburg, Germany) and another assay based on inhibition of FXa (Innovance Antithrombin, Siemens Healthcare Diagnostics Products, Marburg, Germany). AT antigen levels were determined using the Liaphen AT turbidimetric immunoassay (Hyphen Biomed, Neuville-sur-Oise, France). The manufacturer’s stated reference ranges were 79.4–112.0% for the thrombin-based assay, 82.9–118.2% for the FXa-based assay and >80% for the antigen assay.

To monitor thrombin inhibition kinetics, human or bovine α-thrombin (1 ng/mL = 3.94 mU/mL final concentration) was added to citrated plasma. Thrombin inactivation was quenched over time through addition of the reversible active site inhibitor argatroban (200 µmol/L final concentration). The zero time point contained argatroban prior to the addition of plasma. Subsequently, the concentration of argatroban-complexed thrombin representing the free form of thrombin was measured using the thrombin-OECA, that has been initially described by Müller et al. [13] and applied in other studies [11,29,30,31,32,33]. In brief, Maxisorp Fluoronunc microtiter modules were initially coated with 10 µg/mL of bovine serum albumin (BSA, 100 µL/well). After incubation at 4 °C overnight, wells were washed, and a solution of 10 µg/mL streptavidin added and incubated for 1 h at room temperature. Subsequently, wells were washed, blocked with 2% BSA for 2 h at room temperature, and emptied plates stored at −20 °C until used. For running the thrombin-OECA, 1 pmol 3′-biotinylated aptamers HD1–22 were located in the wells (100 µL/well). After washing, plasma samples and calibrators, that covered a ½-log10 concentration range from 0 to 10 ng/mL (0–272 pmol/L) thrombin, were added to the designated wells. After incubation and washing, the fluorogenic thrombin substrate was added. Changes in fluorescence over time were taken as the measure of thrombin captured in the wells. Data obtained from the calibrators were interpolated by 4-parameter curve fit and used to calculate the thrombin concentration in the samples. All samples were assayed in triplicate.

Due to the novelty of our approach of assessment of thrombin inhibition kinetics, there is only limited information about potential confounding factors. Heparin is known to interfere with the thrombin-OECA, and thrombin inhibitors and FXa inhibitors interfere with thrombin-based and FXa-based AT activity assays, respectively [34]. As the focus of this study lay on thrombin inhibition, we excluded patients on heparin or thrombin inhibitors, but not patients on FXa inhibitors, which may have introduced a selection bias. Furthermore, in the patients receiving FXa inhibitors residual drug levels may have interfered with the FXa-based measurement of AT activity.

### 4.4. Statistical Analysis

Statistical analyses were performed using XLSTAT software (Addinsoft, Boston, MA, USA) [35]. Data are generally presented as mean and standard deviation. Normality of data was tested using the Shapiro–Wilk test. As measured half-life times were normally distributed, an ANOVA followed by a paired or unpaired Student *t* test was performed to compare data between cohorts or between experiments with bovine and human thrombin. For the comparison between the three cohorts’ healthy controls, experiments with human thrombin, and experiments with bovine thrombin the Bonferroni correction was performed for three comparisons. Depending on the normality of data Pearson’s correlation coefficient or Spearman’s rank correlation coefficient was used to assess correlations.

## Figures and Tables

**Table 1:** Characteristics of the study subjects with antithrombin mutations.

	Properties	Human Thrombin Inhibition Assessed	Bovine Thrombin Inhibition Assessed
Demographics	Mean age (range), years	35 (4–82)	34 (4–71)
	Males/females	12/24	5/14
Thrombosis history of study subjects ^1^	Asymptomatic subjects	11	4
	Deep vein thrombosis	19	13
	Pulmonary embolism	11	5
	Other venous thrombosis	5	4
	Arterial thrombosis	2	1
*SERPINC1* mutations ^2^	c.89T>A	3	-
	c.391C>T (thereof homozygous)	11 (5)	6 (3)
	c.462_464del	3	3
	c.1058C>T	2	2
	c.1153G>A	3	2
	c.74G>A, c.236G>A, c.470A>G, c.481C>T, c.1347del G, c.1355T>C	1 each	1 each
	c.248T>A, c.331T>A, c.379T>C, c.569A>C, c.721G>T, c.805G>A, c.1246G>T, exon 1–7 large deletion	1 each	-

^1^ The numbers indicate the number of study subjects with at least one of the listed events, in whom thrombin inhibition kinetics were assessed using human thrombin or bovine thrombin, respectively. ^2^ Nucleotide exchanges are described according to HGVS nomenclature. The numbers indicate the number of carriers of the listed mutations. Except for c.391C>T, all carriers were heterozygous.

**Table 2:** Plasma half-life of human or bovine thrombin according to antithrombin mutation.

Nucleotide Exchange ^1^	Amino Acid Exchange ^1^	Antithrombin Activity, Thrombin-Based ^2^	Half-Life of Human Thrombin ^2^	Half-Life of Bovine Thrombin ^2^
c.236G>A	p.Arg79His	100%	50.2 s	101.2 s
c.1246G>T	p.Ala416Ser	77%	62.3 s	-
c.74G>A	p.Gly25Asp	66%	69.8 s	181.1 s
c.805G>A	p.Glu269Lys	62%	75.7 s	-
c.1347del G	p.Leu449fsX9	81%	87.5 s	195.0 s
c.462_464del	p.Phe155fs	52 (52–53)%	92.3 (83.8–107.0) s	168.5 (148.1–184.5) s
c.1355T>C	p.Ile452Thr	84%	98.8 s	117.9 s
c.391C>T	p.Leu131Phe	77 (71–84)%	104.9 (60.6–137.4) s	207.3 (134.3–255.5) s
c.248T>A	p.Leu83Gln	61%	106.1 s	-
c.1153G>A	p.Gly385Ser	58 (57–59)%	106.7 (85.5–123.6) s	174.1 (151.6–196.6) s
c.721G>T	p.Glu241Ter	52%	109.1 s	-
c.89T>A	p.Val30Glu	77 (76–79)%	119.7 (92.7–153.3) s	-
c.1058C>T	p.Pro353Leu	58 (56–60)%	135.6 (106.7–164.5) s	272.2 (197.4–347.0) s
c.481C>T	p.Arg161Ter	51%	139.2 s	203.8 s
c.470A>G	p.Lys157Arg	59%	140.0 s	169.7 s
Exon 1–7 large deletion	-	54%	154.5 s	-
c.391C>T (homozygous)	p.Leu131Phe	55 (47–60)%	154.7 (125.9–177.6) s	263.4 (229.0–331.7) s
c.569A>C	p.Tyr190Ser	63%	164.3 s	-
c.379T>A	p.Cys127Arg	51%	206.6 s	-
c.331T>A	p.Ser111Thr	65%	221.7 s	-

^1^ Nucleotide and amino acid exchanges are described according to HGVS nomenclature. Mutations are sorted in order of ascending plasma half-life of human thrombin. ^2^ Data are presented as means and range, if the plasma half-life has been determined in two or more carriers of the listed mutations. Measurements of antihrombin activity within reference range (79–112%) or human thrombin half-life within the 95th percentile of the healthy control group (human thrombin, <73.2 s; bovine thrombin, <156.3 s) are underlined.

## Data Availability

The data presented in this study are available in this article and the accompanying Supplementary Materials.

## Supplementary Materials

The following are available online at https://www.mdpi.com/1422-0067/22/4/2119/s1, Table S1: AT mutation carriers.
